# ETH3/482: Studies Evaluating Physician-to-Patient Tele-advice Given via Electronic Mails

**DOI:** 10.2196/jmir.1.suppl1.e43

**Published:** 1999-09-19

**Authors:** G Eysenbach, TL Diepgen

**Affiliations:** ^1^Institute for Clinical Social Medicine, HeidelbergGermany

**Keywords:** Quality of Internet Information, Patient-Physician Relationship, Email, Ethical Codes

## Abstract

**Introduction:**

Patients increasingly use the Internet to solicit "tele-advice" via email from physicians. In two different studies we evaluated the quality of the advice given and determined the attitude of physicians and the ethical codes towards giving such tele-advice.

**Methods:**

In both studies, we posed as a patient describing a medical problem (herpes zoster under immunosuppresion) in lay terms and asked physicians for their advice via email. In the first study, we contacted 17 professional cyberdocs who explicitly offered to give tele-advice. In the second study, we sent an "unsolicited" patient email to 58 physicians who did not offer tele-advice to evaluate their reaction. The second study was also complemented by a questionnaire asking them for their attitude towards answering medical questions via email. Finally, we conducted a review of the ethical, legal and professional codes in the US, Germany, Switzerland to investigate differences in the ethical assessment of giving advice via telecommunication in the absence of a pre-existing patient-physician relationship.

**Results:**

From the 17 professional "cyberdocs", only 7 replied giving detailed advice, 2 of the responses were medically wrong. In the second study with the "unsolicited email", 29 (50%) responded to the fictitious patient request; 9 respondents (31%) refused to give advice without having seen the lesion, 27 (93%) recommended that the patient see a physician, and 17 (59%) explicitly mentioned the correct "diagnosis" in their reply. In response to the questionnaire, 8 (28%) of the 29 respondents said that they tended not to answer any patient e-mail, 7 (24%) said they usually reply with a standard e-mail message, and 7 (24%) said they answer each request individually. Slight differences in the professional codes between the US, Germany and Switzerland exist: While the US AMA ethical guidelines only warn to make a diagnosis and considers drug prescriptions unethical, the German code disallows any advice via mail or email in the absence of a physician-patient relationship.

**Discussion:**

These pioneering studies give important hints on the possible economics, problems and ethics of patient-physician communication via email. Responses of physicians and Web masters to e-mail requests for medical advice vary as do approaches to handling unsolicited e-mail. International standards for physician response to unsolicited patient e-mail are needed.

